# Potential role of *Citrus bergamia* flower essential oil against oral pathogens

**DOI:** 10.1186/s12906-024-04457-7

**Published:** 2024-04-12

**Authors:** Muhammad Imran Aziz, Muhammad Mohtasheemul Hasan, Riaz Ullah, Ahmed Bari, Mohsin Abbas Khan, Syed Zia Ul Hasnain, Rabia Baloch, Muhammad Akram, Aqsa obaid, Aziz Ullah, Khizar Abbas, Adnan Amin

**Affiliations:** 1 Department of Pharmacognosy, Faculty of Pharmacy and Pharmaceutical Sciences, University of Karachi, Karachi, 75270 Pakistan; 2 Department of Pharmacognosy, College of Pharmacy, King Saud University, Riyadh, Saudi Arabia; 3 Department of Pharmaceutical Chemistry, College of Pharmacy, King Saud University, Riyadh, Saudi Arabia; 4 Department of Pharmaceutical Chemistry, Faculty of Pharmacy, The Islamia University of Bahawalpur, Bahawalpur, Pakistan; 5 Department of Pharmacognosy, Faculty of Pharmacy, Bahauddin Zakaria University, Multan, Pakistan; 6 Allama Iqbal Teaching Hospital, Dera Ghazi Khan, Pakistan; 7 Pakistan Council for Scientific and Industrial Research (PCSIR), Peshawar, Pakistan; 8 NPRL, Department of Pharmacognosy, Faculty of Pharmacy, Gomal University, 29050, D.I. Khan, Pakistan; 9 Department of Chemistry, Qurtaba University, D.I.Khan Campus, D.I.Khan, Pakistan; 10 Pukyong National University, Yangso-Ro, 48513 Busan, Republic of Korea

**Keywords:** Cell to cell signaling, Oxidative stress, Aroma, In silico analysis, Drug resistance

## Abstract

**Background:**

Oral bacterial infections are difficult to treat due to emergence of resistance against antibiotic therapy. Essential oils are considered emerging alternate therapy against bacterial infections and biofilms. We investigated *Citrus bergemia* flower essential oil against oral pathogens.

**Methods:**

The essential oil was analsyed using Gas Chromatography(GC–MS), in silico investigations, antioxidant, antimicrobial, antibiofilm and antiquorum sensing assays.

**Results:**

Gas Chromatography analysis confirmed presence of 17 compounds including 1,6-Octadien-3-ol,3,7-dimethyl, 48.17%), l-limonene (22.03%) and *p*-menth-1-ol, 8-ol (7.31%) as major components. In silico analysis showed compliance of all tested major components with Lipinski’s rule, Bioavailability and antimicrobial activity using PASS (prediction of activity spectrum of substances). Molecular docking with transcriptional regulators 3QP5, 5OE3, 4B2O and 3Q3D revealed strong interaction of all tested compounds except 1,6-Octadien-3-ol,3,7-dimethyl. All tested compounds presented significant inhibition of DPPH (2,2-diphenyl-1-picrylhydrazyl) (IC_50_ 0.65 mg/mL), H_2_O_2_ (hydrogen peroxide) (63.5%) and high FRAP (ferrous reducing antioxidant power) value (239.01 µg). In antimicrobial screening a significant activity (MIC 0.125 mg/mL) against *Bacillus paramycoides* and *Bacillus chungangensis* was observed. Likewise a strong antibiofilm (52.1 – 69.5%) and anti-QS (quorum sensing) (4–16 mm) activity was recorded in a dose dependent manner.

**Conclusion:**

It was therefore concluded that *C. bergemia* essential oil posess strong antioxidant, antimicrobial and antibiofilm activities against tested oral pathogens.

**Supplementary Information:**

The online version contains supplementary material available at 10.1186/s12906-024-04457-7.

## Background

The Food and Agricultural Organization (FAO) enlists citrus as one of the most important crops with reference to annual production across the globe [1]. Citrus fruits are being used since ancient times for management of several health related problems including scurvy, common cold, menstrual irregularities, myocardial infarction, coronary artery disease and high blood cholesterol due to strong antioxidant potential [2, 3]. Citrus fruits and peel are considered as rich source of diverse flavonoids, polyphenolic compounds, carboxylic acids, vitamin C and amino acids [4, 5]. Amongst all *Citrus bergamia* (Rutaceae, *C. bergemia*) has gained great attraction from researchers because of its peculiar health benefits [6]. *C bergemia* is native to Italy and Spain however it is cultivated throughout the world [7]. The geographical and botanical origins of this particular fruit are still uncertain [8].

Bergamot contains essential oils in peel and flowers that has largely been used in medical, food, cosmetics, perfumes, and confectionary industry [3]. The essential oils (EOs) are complex mixtures of chemical compounds, each having a unique intense odor, and are located in different parts of the plants including seeds, fruits, stems, leaves, roots, and flowers [9]. The EOs are the secondary metabolites formed as a result of plant defensive mechanisms through the involvement of enzymes [10]. It has been emphasized that EOs are the final terpenoid products produced in plants as a result of enzymes like terpene synthase [11]. Numerous studies have reported the therapeutic potential of the EOs including antimicrobial, anticancer, and antioxidant properties. Being an antimicrobial moiety, EOs are also used to preserve food items [12]. Bergemia essential oil (BEO) is traditionally used for the treatment of parasitic infections, sore throat, tonsillitis, wound healing, hyperhidrosis, vaginal pruritis, leucorrhoea, skin and mouth infections, gonococcal infections, urinary tract and respiratory tract infections [13].

Oral complications including dental abscess, gingivitis, dental caries and periodontitis are mainly due to oral bacterial infections [14, 15]. These infections are main reason for global health burden ($442 billion) [16] and considered serious since these can lead to cancer and death in worst conditions [17]. It has been estimated that a huge population (3.5 billion) of the world is currently effected by oral diseases untreated dental caries is one of the most common non-communicable disease [14]. The treatment failures and development of antibiotic resistance [18] has and the importance of natural origin antimicrobials motivated us to explore the effect of the bergemia flower essential oil (BFEO) effect against oral microorganisms. The core aim of the study was to investigate BFEO against oral pathogens using in silico and in vitro models.

## Methods

### Plant material and isolation of essential oil

The fresh flowers of *Citrus bergemia* were collected with consent from farm house in Paharpur area (District D.I.Khan, KPK, Pakistan) and authenticated by Dr. Zain Ul Abedene (Taxonomist in Institute of Biosciences, Gomal University D.I.Khan, Pakistan). The collected flowers (50 g) were stored at -40 °C for essential oil extraction in clevenger- type apparatus to isolated essential oil using hydro-distillation. The anhydrous sodium sulphate (anhydrous) was used for drying of collected essential oil and removal of water. Finally, essential oil was stored at 4 °C.

### Microbial strains and growth media

The clinical strains were isolated from dental plaque of female diabetic patients with the help of Dentist. Strains were transferred to lab and purifications, isolation of strains was performed using differential media including EMB (eosin methylene blue) agar, Mackonkey agar and congo red agar. The strains were identified by 16S rRNA as *Bacillus chungangensis, Bacillus paramycoides, Bacillus chungangensis, Paenibacillus dendritiformis, Staphylococos aureus and Staphylococus epidermidis.* Standard bacterial strains were *E.coli* (ATCC 25922), *Staphylococcus aureus* (ATCC 33862)*, Klebsiella pneumoniae* (ATCC BAA-1705). Biomarker strains included *Chromobacterium violaceum* (DSM 30191). Growth and differential media used during the investigation including Lauria bertani (LB), BHIA (brain heart infusion agar), tryptic soya broth (TSB) were purchased from Hi Media (India) while Mackonkey agar and eosin methylene blue agar was purchased from (Oxoid, UK).

### GC–MS analysis

Essential oil component analysis was accomplished by means of GCMS (Shimadzu GC 2010, Japan) having installed, auto sampler (AOC-20i autosampler) and suitable capillary column (dimensions 30 m × 0.25 mm id, 0.25 μm film thickness, a DB-5 MS column). System oven temperature was set (initially at 45–90 °C) with a rise rate of 2 °C (per min), then increase from 91 to 240 °C with a rise rate of 3 °C (per min). Finally achieved temperature (240 °C) was set constant for 5 min. The temperature of injector (240 °C) and detector (280 °C) was kept constant at set temperatures. For loading the sample, an aliquot of essential oil was (0.5 μL) was injected and Helium (1 ml/min) was used as a carrier gas. GCMS component analysis and identification was accomplished on a GCMS-QP 2010 Plus (Shimadzu, Japan) system operating in EI mode (electron ionization mode) at 70 eV. Mass units were monitored from 35 to 500 AMU. The NIST mass spectral library and compound mix (including limonene, carvacrol, thymol, α-pinene, β-pinene, β-myrcene and sabinene) was used for identification of compounds [19].

### Drug likeness, PASS and bioavailability (Lipinski properties)

On relative abundance basis, following major compounds were further chosen for drug likeness and bioavailability and PASS including 1,6-Octadien-3-ol,3,7-dimethyl(linalool) (**1**) L-limonene (**2**); *p*-menth-1-ol,8-ol (**3**); aromadendrine (**4**); β-myrcene (**5**) and β-pinene (**6**). The Lipinski properties [20], bioavailability and PASS analysis were performed using molinspiration tool [21] SWISS ADME [22, 23] and ways2 drugs tool [24].

### Molecular docking

For Molecular docking studies, the X-ray crystallographic structures of the transcriptional regulators LasR (2UV0), PqsE (2Q0J) [25] and quorum sensing regulators CviR (3QP5) [26] were obtained from the Protein Data Bank (PDB). The active site dimensions for each protein were recorded by using their co-crystallized ligands respectively. Then, the water molecules and co-crystallized ligand were removed and hydrogen atoms and charges were added. The SDF format for 3D structures of all the phyto-constituents were downloaded from Pubchem database and PDB files were generated in Accelrys DS Visualizer 2.0 (Accelrys Software Inc., 2012). The molecular docking was performed using Lamarckian Genetic Algorithm embedded in AutoDock v 4.2. [27]. A total number of 63 different poses were generated and clustered according to their RMSD values. Each cluster was carefully visualized in Discovery Studio Visualizer [28] and putative binding modes were selected accordingly. Best docked structures based on the binding energy scores (ΔG) were chosen for further analyses. The hydrogen bonding and hydrophobic interactions between ligand and protein were calculated by Accelrys DS Visualizer 2.0 [29].

### Biological activities

#### Antioxidant activities

##### Hydrogen peroxide (H_2_O_2_) assay

A modified method was employed for determination of H_2_O_2_ based antioxidant activity [30]. Concisely, H_2_O_2_ stock solution (2 mM) was prepared followed by mixing (600 µL) with test sample (400 µL). The reaction solution was vortexed and absorbance was measured after 10 min at 230 nm. As reference standard, ascorbic acid was used and results were expressed as below

$$\mathrm{\%\ Inhibition }= (1- {{\text{A}}}_{0} /{{\text{A}}}_{1})\times 100$$

where A_0_ is absorbance of sample and A1 was absorbance of sample.

##### DPPH Assay

The antioxidant activity of samples was determined by using modified procedure [31]. Briefly, DPPH (freshly prepared) solution in methanol (0.5 mL; 0.1 mM) was combined with plant extract (0.5 mL, several concentrations) and placed 30 min (In dark place to avoid light effect). Later absorbance was observed at 517 nm. As reference standard, ascorbic acid was used. Results were expressed as below;

$$\mathrm{\%\ inhibition }= (1-\mathrm{ Absorbance\ of\ test\ sample}/\mathrm{Absorbance\ of\ reaction\ control}) \times 100$$

A linear plot was used to calculate IC_50_ values of test samples.

##### FRAP assay

Ferric ion reducing power of tested sample was determined using modified method [32]. Concisely, fresh FRAP reagent was set by adding TPTZ (10 mM prepared in 40 mM HCL) to acetate buffer (300 mM), and ferric chloride (20 mM) in specified ratio (10:1:1). Tested sample (100 µL) was reacted with FRAP reagent (3 mL) and placed in dark place (30 min). Afterwards, absorbance was recorded (at 593 nm) in UV–Vis spectrophotometer. The ferrous reducing capacity was determined by FeSO_4_ standard curve and results were expressed as µg equivalents.

#### Antimicrobial activities

##### Determination of MIC

The isolated compounds were assessed for antimicrobial activities using modified method [33] with slight modifications. In the MIC assay, the 96 microwell plates were loaded with 50 µL of the overnight-grown bacterial strain (1.5 × 10^7^ CFU/mL), followed by addition of 50 µL of test sample (various dilutions). The plates were incubated at 37 °C for 24 h. On the next day, 40 µL of resazurin solution (0.015%) was added to each well followed by incubation at 37 a °C for further 60 min. Colorimetric readings were recorded using 96-microplate reader (Hippo MPP-96, Biosan). For MBC values, bacterial suspensions (10 μL) from the MIC microwell were relocated to already prepared agar plates (Muller Hinton) and incubated for 24 h. Afterwards, bacterial growth was recorded on the agar plates. All samples were loaded in triplicate. Ciprofloxacin was used as positive control.

##### Antibiofilm activity

The biofilm formation assay was performed using 12-well polystyrene plates with a slightly modified method [34]. Briefly, the bacterial strain was inoculated in TSB medium (280 μL) at an initial turbidity of 0.5 at 600 nm (0.5 McFarland). And allowed to incubate for 24 h to produce biofilm. Afterwards 100 μL of test compound (0.01–3 mg/mL) was added to the bacterial culture followed by incubation at 37 °C for further 24 h. Cell growth in the plates was measured at 592 nm. For quantification, the biofilms in the 12-well plates were stained using crystal violet. Afterwards 95% ethanol was added to the stained cells and absorbance was recorded at 592 nm to quantify total biofilm formation.

The % inhibition was calculated using following formula

$$\mathrm{\%\ inhibition }= (1-\mathrm{ Abs\ of\ sample}/\mathrm{Abs\ of\ control }\times 100)$$

##### Antiquorum sensing

The quorum sensing inhibition potential of isolated compounds was evaluated by a standard procedure [34] with slight modifications. An overnight culture of *Chromobacterium. violaceum* (1/100 ratio) was streaked onto LB agar in Petri dishes. Sterilized filter paper discs (6 mm) were prepared and placed on the top of BHIA (Brain Heart Infusion agar) seeded with indicator strain (*C. violaceum*). Then 15µL test compound (0.01–3 mg/mL) was applied on each disc and allowed to dry for 30 min. Afterwards, the assay plates were incubated at 30 °C for 3 days. Ciprofloxacin was used as standard drug. Finally, results were recorded by measuring the zone of inhibition around each disc.

### Violacin inhibition assay

A modified method [34] was adopted for violacein inhibition assay. A 24 h old culture (200 μL of *C. violaceum* (OD = 0.4 OD at 600 nm) was loaded to sterilized microtiter plates containing various concentrations of compounds (1–4 mg/mL). The plates were incubated at 30 °C for 24 h and witnessed for the decrease in violacin pigment production by taking absorbance at 585 nm. The percentage inhibition was calculated by following the formula:

$$\mathrm{Violacein\ inhibition\ \% }= (1-\mathrm{ Absorbance\ of\ sample }/\mathrm{Absorbance\ of\ control }\times 100)$$

### Statistical analysis

All biological activity experiments were performed in three independent experiments data was expressed as ± SD. One way ANOVA followed by post-hoc Tukey test with *p* < 0.05.

## Results

### GC–MS analysis

**Fig. 1:**
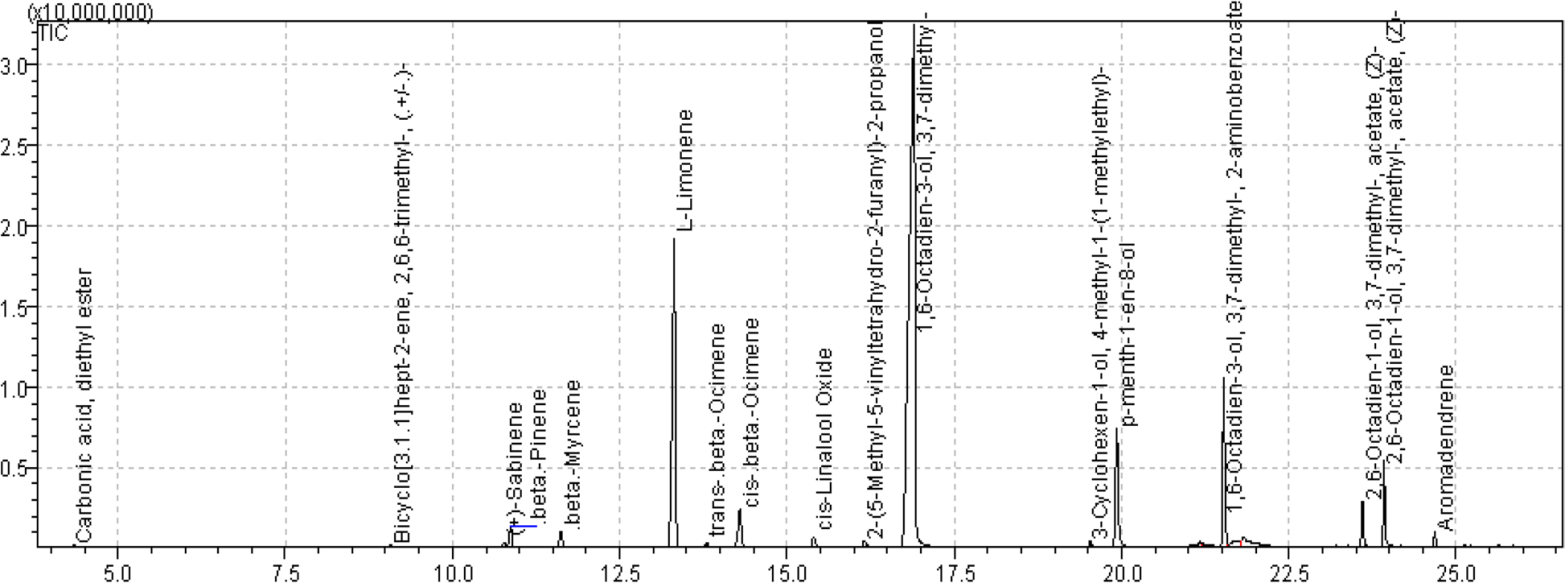
GCMS analysis of *Citrus bergemia* essential oil

**Fig. 2:**
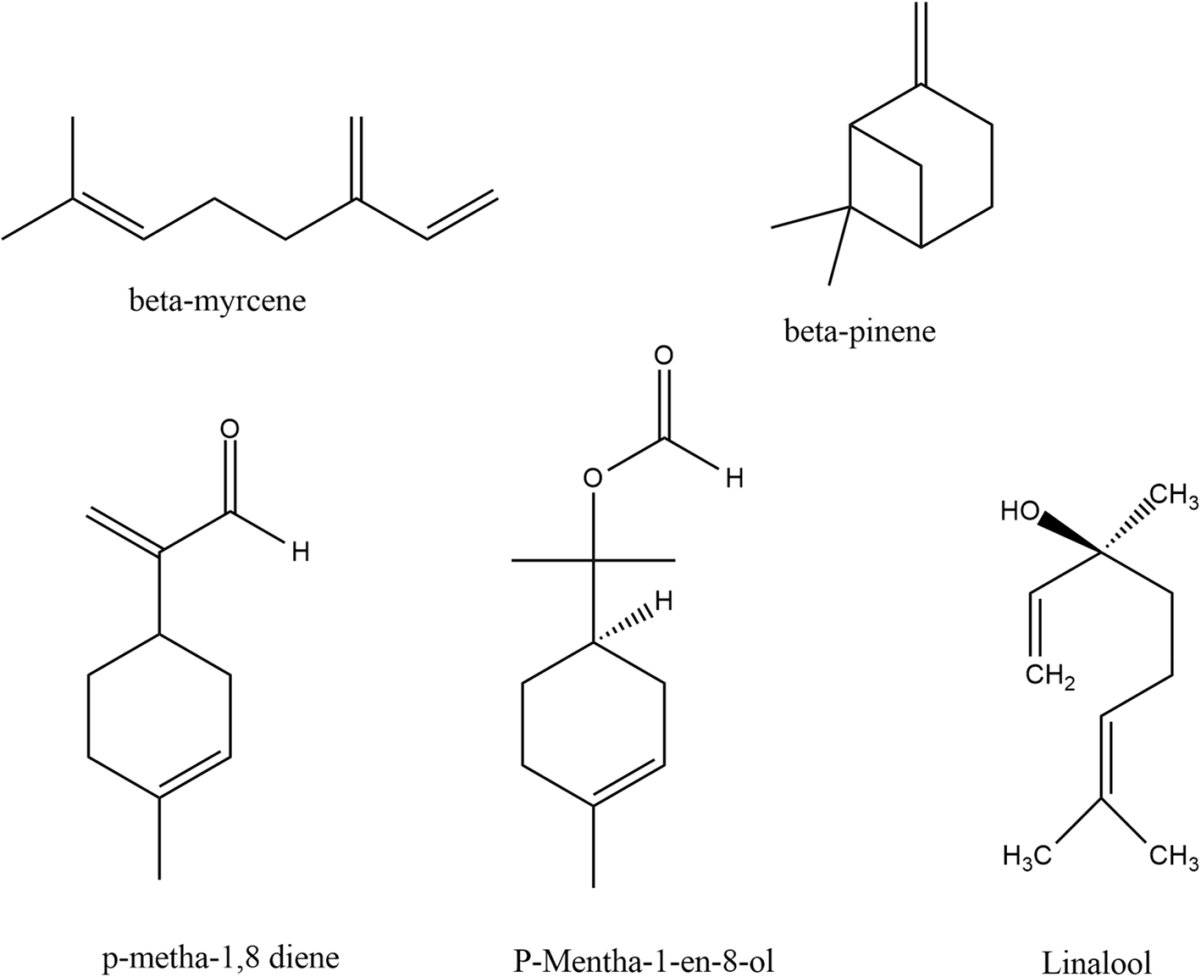
Structure of major components detected in *C. bergemia* essential oil

The *C. bergemia* essential oil component analysis was accomplished by using GC–MS analysis. A total of 17 compounds were identified with the help of standard mix and NIST libraries. linalool (1,6-Octadien-3-ol,3,7-dimethyl) was reported with highest concentration (48.17%, RT 19.898 min) (Fig. 1, Table 1). Other major components included l-limonene (22.03%; RT 13.315 min), *p*-menth-1-ol, 8-ol (7.31%, 19.929 min). Components present in minor concentration included sabinene (0.46%, 10.771 min), β-pinene (1.69%, RT 10.870 min), β-myrcene (1.57%,11.622 min) and aromadendrine (0.36%, 24.683 min) (Fig. 2).

**Table 1:** GC–MS Profile of *Citrus bergemia* flower essential oil

S. No	Name	R.Time	Area	Conc %	KI^a^
1	carbonic acid, diethyl ester	4.340	194,238	0.40	903.5524
2	bicyclo[3,1,1]hept-2-ene,2,6,6-trimethyl-,(. ±)-	9.084	129,269	0.27	1060.2445
3	( +)-sabinene	10.771	225,590	0.46	1100.8982
4	β-pinene	10.870	819,042	1.69	1103.1659
5	β-myrcene	11.622	762,837	1.57	1120.5924
6	l-Limonene	13.315	10,693,050	22.03	1153.4378
7	trans-.beta.-Ocimene	13.805	175,832	0.36	1162.3927
8	cis-,beta,-Ocimene	14.290	1,227,450	2.53	1170.9485
9	cis-Linalool Oxide	15.400	333,399	0.69	1208.5108
10	2-(5-Methy-5-vinyltetrahydro-2-furanyl)-2-propanol	16.163	192,099	0.40	1226.7358
11	1,6-Octadien-3-ol,3,7-dimethyl-	19.898	23,376,420	48.17	1243.5858
12	3-cyclohexen-1-ol,4-methyl-1-(1-methylethyl)	19.532	142,976	0.29	1297.8606
13	p-menth-1-ol,8-ol	19.929	3,549,232	7.31	1307.5465
14	1,6-Octadien-3-ol,3,7-dimethyl-,2-aminobenzoate	21.533	3,015,202	6.21	1344.8089
15	2,6-Octadien-1-ol,3,7-dimethyl,acetate,(z)-	23.603	1,066,274	2.20	1388.9916
16	2,6-Octadien-1-ol,3,7-dimethyl-,acetate,(z)-	23.927	2,452,949	5.05	1395.5544
17	Aromadendrene	24.683	173,862	0.36	1413.1359

^a^Kovats retention index

### Drug likeness, bioavailability and PASS analysis

**Fig. 3:**
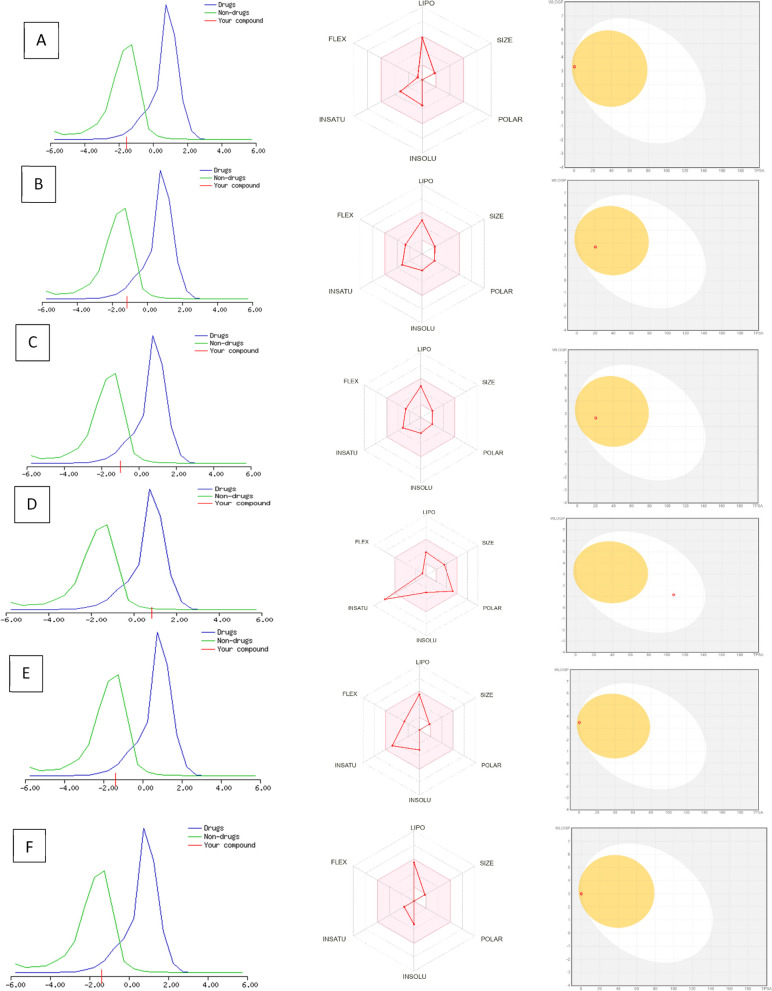
Druglikeness scores, Bioavailability radar and boiled egg model for major components in *C. bergemia* limonene (**A**), P-menth-1-en-8-ol (**B**), linalool (**C**), Aromadendrin (**D**) β- myrecine (**E**) and Pinene (**F**)

Major identified components of *C. bergemia* were checked for drug likeness using Lipinski’s rule of five. It was evident that all tested compounds (except β-pinene) showed a compliance with lipinski’s rule and therefore were considered as suitable candidates for further analysis (Table 2). Since Log *p* value of β-pinene (Log p 4.14) showed a slight deviation from set value (Log *p* < 5), a violation was observed. Further, drug likeness scores of l- limonene, *p*-menth-1 en-8-ol, β-myrcene and β-pinene presented a slight deviation from standard value (+ 1 to -1) (Table 2, Fig. 3).

**Table 2:** Lipinski properties of major components in *C. bergemia* flower essential oil

**S. No**	**Compound**		**M. weight** ** < 500 Da**	**Drug likeness** **score**	**Log *p* < 5**	**H Bond Donor(5)**	**H-Bond accepto*r* < 10**	**No of violations**
1	L-Limonene	CC1 = CCC(CC1)C(= C)C	136.13	-1.54	4.53	0	0	0
2	1–6 octa-diene-3-ol, 3,7-dimethyl (linalool)	CC(= CCCC(C)(C = C)O)C	154.14	-0.99	3.07	1	1	0
3	*p*-menth-1-en-8-ol	CC1 = CCC(CC1)C(C)(C)OC = O	182.13	-1.16	3.42	0	2	0
4	aromadendrine	C1 = CC(= CC = C1C2C(C(= O)C3 = C(C = C(C = C3O2)O)O)O)O	288.06	0.85	1.42	4	6	0
5	β-myrcene	CC(= CCCC(= C)C = C)C	136.13	-1.38	4.18	0	0	0
6	β-pinene	CC1(C2CCC(= C)C1C2)C	136.13	-1.39	4.14	0	0	1

**Fig. 4:**
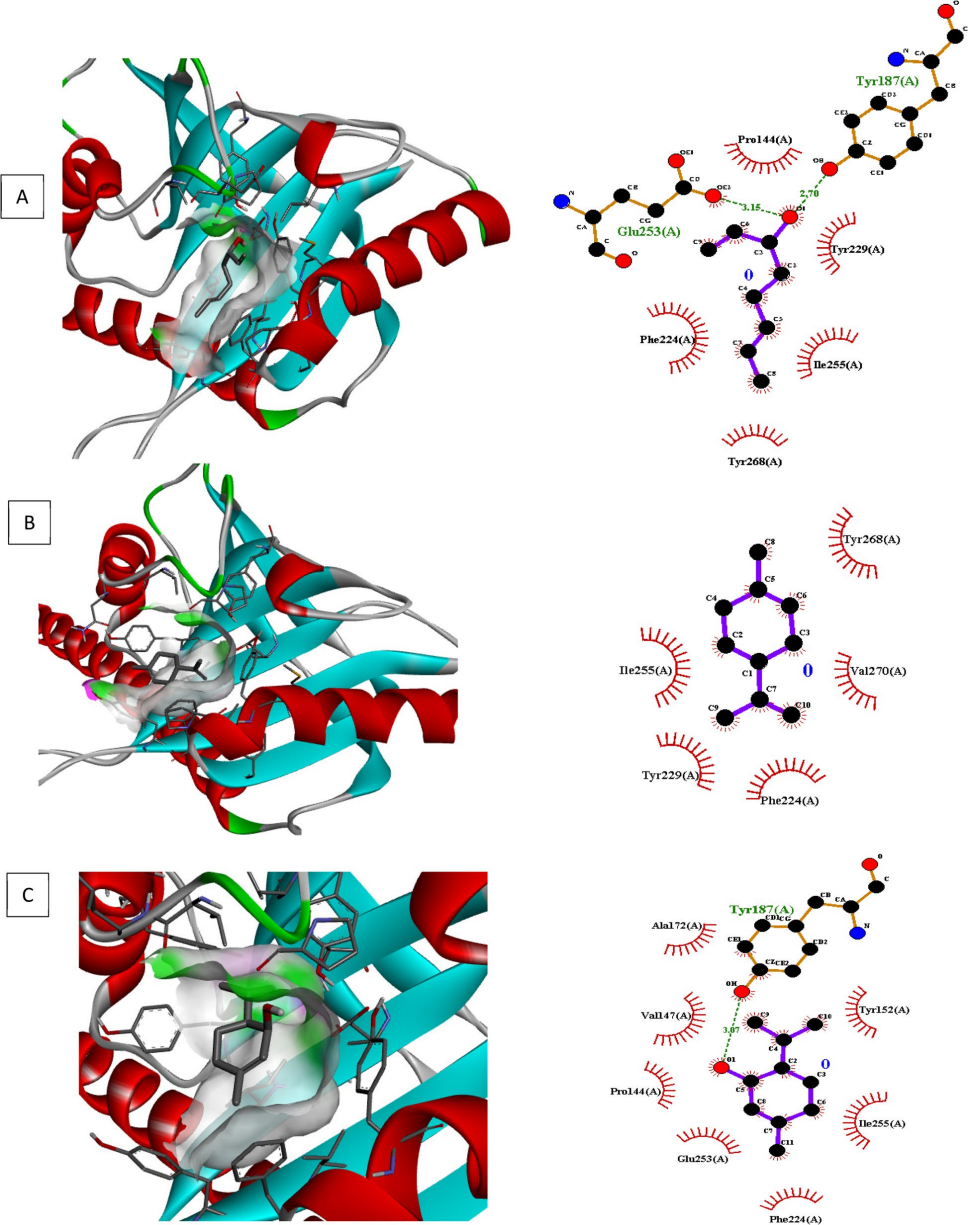
Molecular docking, interaction analysis of Linalool (**A**, Pose 1), Limonene (**B**, Pose 1) and P-menth-1-en-8-ol (**C**, Pose 3) against 3Q3D

The pharmacokinetic parameters were evaluated by using SWISS ADME (Table 3). It was evident from boiled egg model that all tested components were able to cross blood brain barrier (BBB) except aromadendrine. Whereas Bioavailability radar showed that limonene, β-myrcene had low absorbtion from GIT. Further, all parameter (insaturation, polarity, Molecular weight, insolubility, flexibility and lipophilicity) of bioavailability radar were with permissible limits for tested compounds except for aromadendrine with a slight increase in case of in saturation (Fig. 4). PASS analysis revealed that all tested compounds had a potential to interact with bacterial cells (0.4973 – 0.6049 confidence interval) (Table 4). Therefore these potential drug candidates were further processed for selective molecular docking analysis.

**Table 3:** Pharmacokinetic parameter of major components from *C. bergemia* using SWISS-ADME tool

S.No	Compounds						
	Parameter	limonene	1–6 octa-diene-3-ol, 3,7-dimethyl (linalool)	p-menth-1-en-8-ol	aromadendrine	β-Myrcine	β-pinene
1	GIT Absorbtion	Low	High	High	High	Low	Low
2	BBB permeant	Yes	Yes	Yes	No	Yes	Yes
3	P-gp substrate	No	No	No	No	No	No
4	CYP1A2 inhibitor	No	No	No	No	No	No
6	CYP2C19 inhibitor	Yes	No	No	No	No	No
7	CYP2C9 inhibitor	No	No	Yes	No	No	Yes
8	CYP2D6 inhibitor	No	No	No	No	No	No
9	CYP3A4 inhibitor	No	No	No	No	No	No
10	Log *K*_p_ (skin permeation)	-3.89 cm/s	-5.13 cm/s	-4.59 cm/s	-7.13 cm/s	-4.17 cm/s	-4.18 cm/s

**Table 4:** PASS of major components from *C. bergemia* using online tools (Ways2drugs)

	Sample	Bacterial strain	Confidence
1	limonene	*Salmonella enteritidis*	0.4973
		*Staphylococcus simulans*	0.4038
		*Prevotella oralis*	0.4004
		*Micrococcus luteus*	0.3551
2	1–6 octa-diene-3-ol, 3,7-dimethyl (linalool)	*Staphylococcus simulans*	0.5192
		*Prevotella intermedia*	0.4129
		*Staphylococcus lugdunensis*	0.3922
		*Lactobacillus plantarum*	0.3920
3	*p*-menth-1-en-8-ol	*Staphylococcus simulans*	0.4523
		*Klebsiella pneumoniae*	0.4082
		*Prevotella intermedia*	0.3908
		*Prevotella melaninogenica*	0.3758
4	aromadendrine	*Listeria monocytogenes*	0.6195
		*Pseudomonas fluorescens*	0.4828
		*Bacillus subtilis*	0.4578
		*Mycobacterium intracellulare*	0.3953
5	β-myrcine	*Staphylococcus simulans*	0.6049
		*Prevotella intermedia*	0.4891
		*Listeria monocytogenes*	0.4306
		*Lactobacillus plantarum*	0.3945
6	β-pinene	*Prevotella melaninogenica*	0.5889
		*Prevotella intermedia*	0.5514
		*Streptococcus mutans*	0.5004
		*Fusobacterium nucleatum*	0.4526

### Molecular docking investigations

**Fig. 5:**
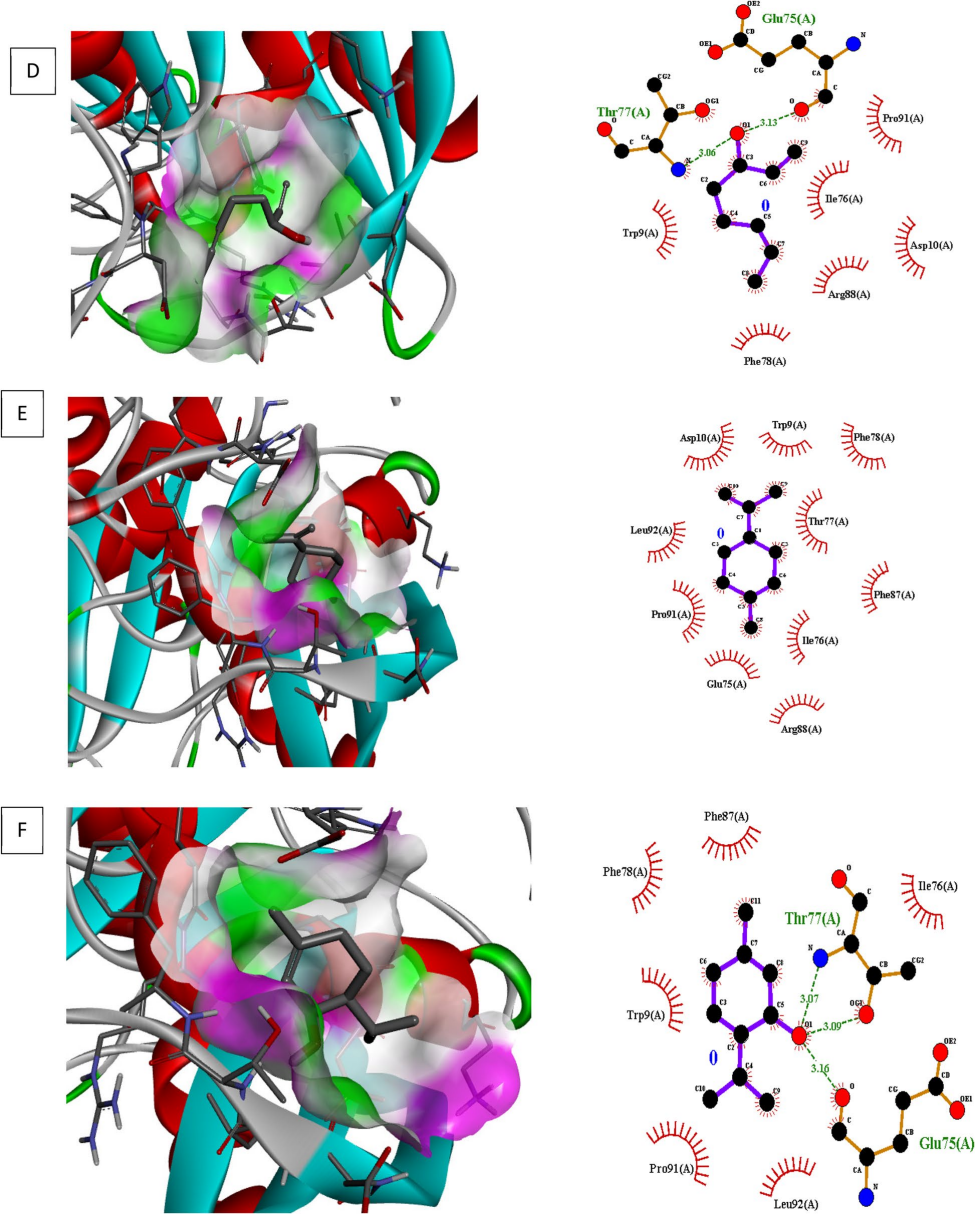
Molecular docking, interaction analysis of Linalool (**D**, Pose 1), Limonene (**E**, Pose 1) and P-menth-1-en-8-ol (**F**, Pose 3) against 4B2O

**Fig. 6:**
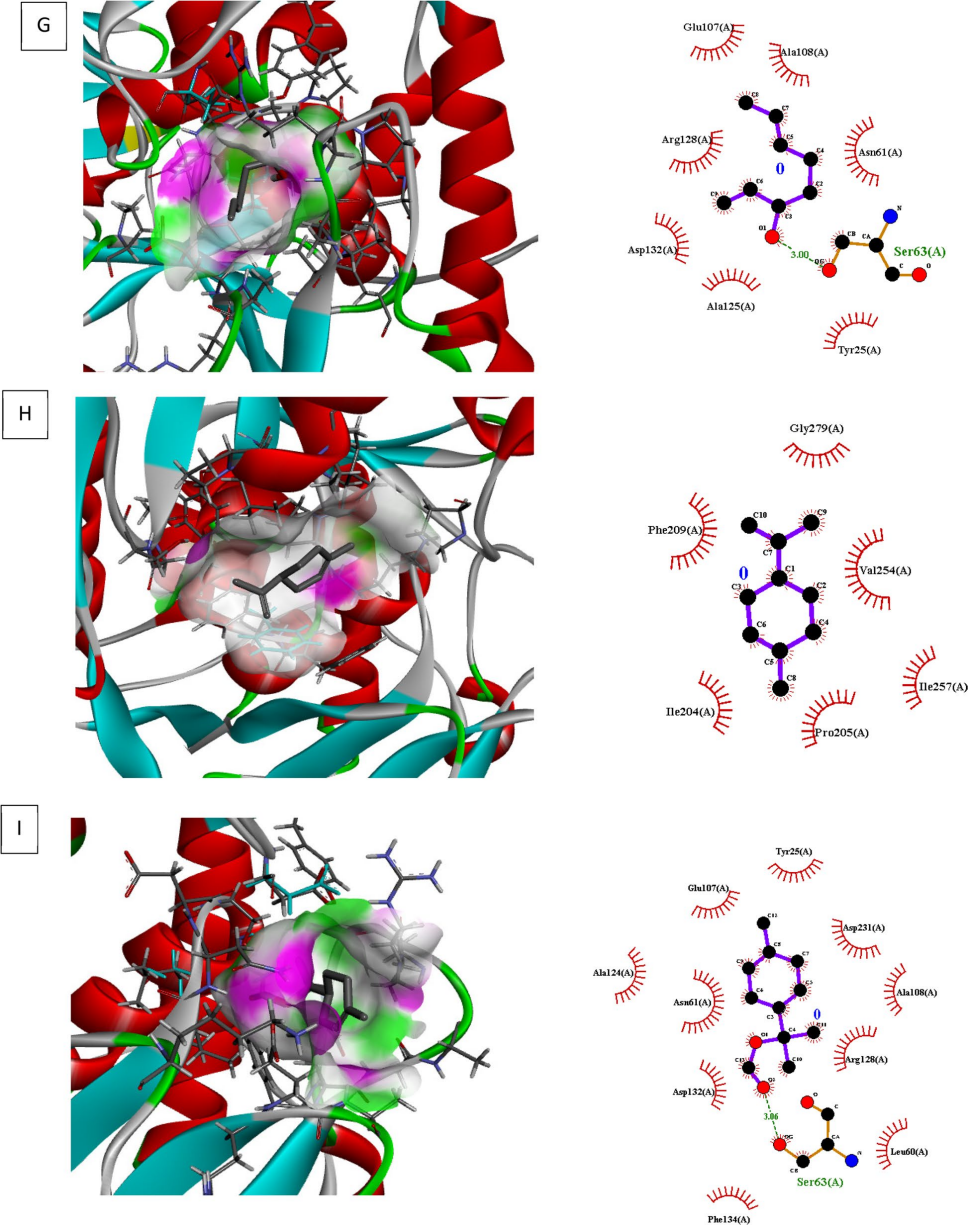
Molecular docking, interaction analysis of Linalool (**G**, Pose 1), Limonene (**H**, Pose 1) and P-menth-1-en-8-ol (**H**, Pose 1) against 5OE3

**Fig. 7:**
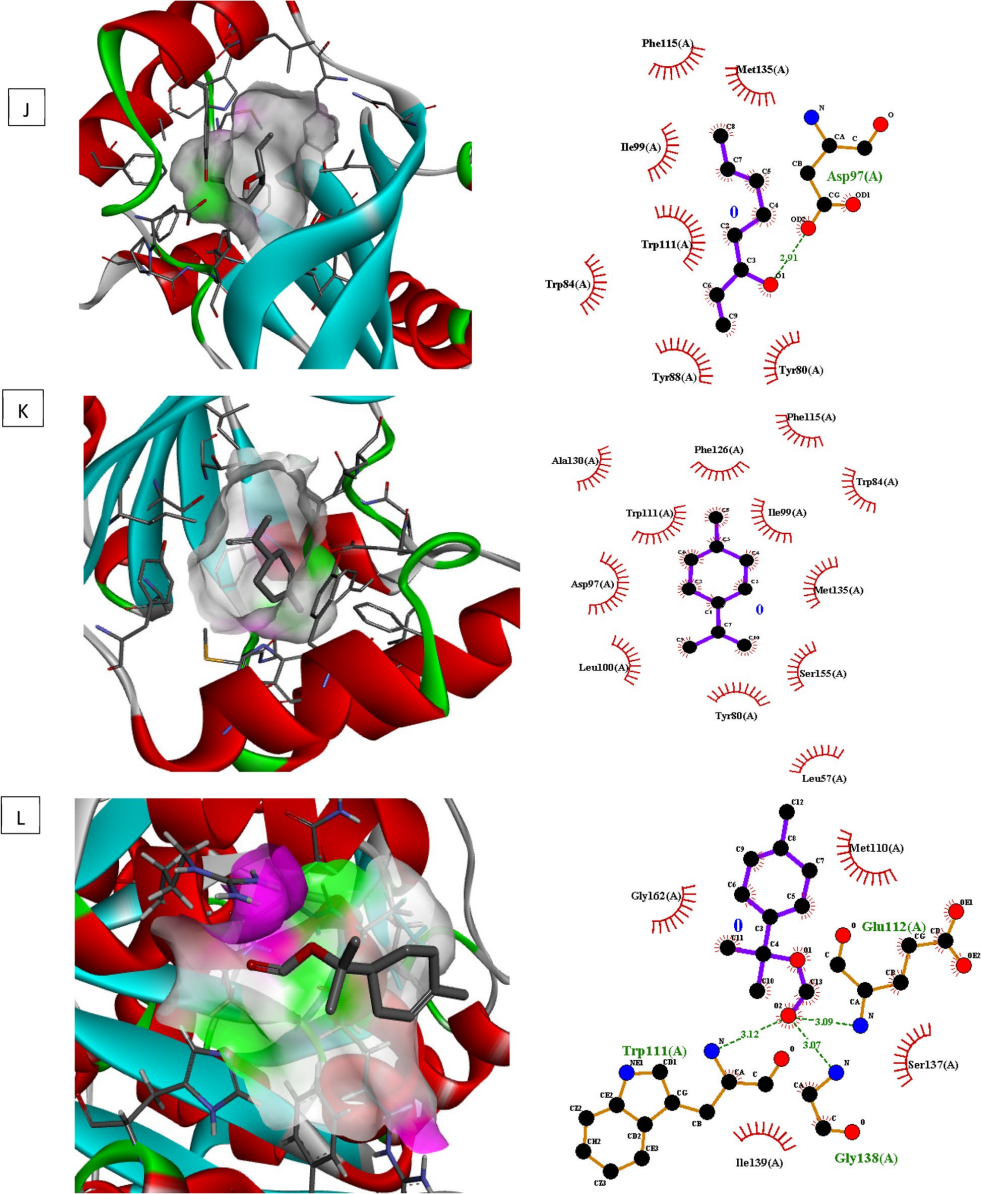
Molecular docking, interaction analysis of Linalool (**J**, Pose 1), Limonene (**K**, Pose 1) and P-menth-1-en-8-ol (**L**, Pose 7) against 3QP5

Molecular docking of different target for biofilm and quorum sensing (3QP5, 5OE3, 4B2O and 3Q3D) was performed using selected ligands (L-limonene, linalool, and p-menth-1 en-8ol). In interaction analysis with regulator gene 3Q3D, *p-*menth-1 en-8ol presented strong interaction ((-5.2 ΔG (kJ mol^‒1^) with Glu253 and Tyr187 via H-bonding whereas neighboring amino acids included Tyr229, Ile255, Tyr268, Phe224 and Pro144 (Table 5, Fig. 4).In case of transcriptional regulator 4B2O, both linalool and *p*-menth-1 en-8ol showed significant H–bonding interaction with target. Based on free binding energy, *p*-menth-1 en-8ol (-5.7ΔG (kJ mol^‒1^) was able to show specific H-bonding interactions with amino acid Thr77 and Glu 75. In this case nearby interaction residues were Leu92, Pro91, Trp9, Phe78 and Ile76 (Fig. 5, Table 5). In 5OE3, both linalool and p-menth-1 en-8ol were able to interact with Ser63 amino acid residue, however the hydrophobic interactions were greater in case of *p*-menth-1 en-8ol that included Phe134, Asp132, Asn61, Ala124, Glu107, Try25, Asp231, Ala108, Arg128, and Leu60 amino acid residues with high free energy ((-6.1 ΔG (kJ mol^‒1^) on pose 1 (RMSD 0) (Fig. 6, Table 5). In case of 3QP5, p-menth-1 en-8ol showed specific H- bonding (-5.0 ΔG (kJ mol^‒1^) interactions with Gly138, Trp111 and Glu 112, where the hydrophobic (weak) interactions were seen case of Ile139, Gly162, Met110, Ser137 (Fig. 7, Table 5). Linalool was not able to show any H-bonding interaction with any of transcriptional regulator, however, it participated in hydrophobic interactions (Table 5).

**Table 5:** Docking score and interaction analysis of major components in *C. bergemia*

Ligand	Binding free energy ΔG (kJ mol^‒1^)	Pose rank	No of H bonds	H Bond Interaction Residues	Other interaction residues
**3QP5**					
l-limonene	-5.5	1	0	0	Tyr80, Leu100, Asp97, Trp111, Ala130, Phe126, Phe115, Trp84, Met135, Ser155,Leu57
1–6 octa-diene-3-ol, 3,7-dimethyl	-5.2	1	1	Asp97	Tyr80, Tyr88, Trp84, Trp111, Ile99, Phe115, Met135
p-menth-1-en-8-ol	-5.0	7	3	Gly138, Trp111, Glu112	Ile139, Gly162, Met110, Ser137
**5OE3**					
l-limonene	-5.5	1	0	0	Ile204, Phe209, Gly279, Val254, Ile257,Pro205
1–6 octa-diene-3-ol, 3,7-dimethyl	-5.2	1	1	Ser63	Tyr25, Ala125, Asp132, Arg128, Glu107, Ala108, Asn61,
p-menth-1-en-8-ol	-6.1	1	1	Ser63	Phe134, Asp132, Asn61, Ala124, Glu107, Try25, Asp231, Ala108, Arg128, Leu60
**4B2O**					
l-limonene	-5.2	1	0	0	Arg88, Glu75, Pro91, Leu92, Asp10, Trp9, Phe78, Thr77, Phe87, Ile76,
1–6 octa-diene-3-ol, 3,7-dimethyl	-4.5	1	2	Thr77, Glu75	Pro91, Ile76, Asp10, Arg88, Phe78, Trp9,
p-menth-1-en-8-ol	-5.7	2	2	Thr77, Glu75	Leu92, Pro91, Trp9, Phe78, Ile76
**3Q3D**					
l-limonene	-6.1	1	0	0	Tyr268, Val270,Phe224, Tyr229,Ile255
1–6 octa-diene-3-ol, 3,7-dimethyl	-5.2	1	2	Glu253,Tyr187	Tyr229, Ile255, Tyr268, Phe224,Pro144,
p-menth-1-en-8-ol	-6.0	1	1	Tyr187	Tyr152, Ile255, Phe224, Glu253, Pro144, Val147, Ala172,

### Antioxidant assays

The antioxidant profile of *C. bergemia* was determined by using DPPH, FRAP and H2O2 assays. The quantitative phytochemical analysis revealed significant phenolic contents (84.2 mg/g). The DPPH assays presented significant antioxidant activity (IC_50_ 0.65 mg/mL), whereas a moderate FRAP value (239.01 µg) and H_2_O_2_ (63.5%) inhibition was recorded (Table 6).

**Table 6:** Antioxidant activity of *C. bergemia* flower essential oil

S. No	Sample name	DPPH IC_50_ mg/mL	H_2_O_2_ % inhibition^a^	FRAP value µg	Total Phenolics (mg/g)
1	essential oil	0.65 ± 0.01	63.5 ± 1.2	239.01 ± 2.3	84.2 ± 0.48
2	Ascorbic Acid	17.2 ± 0.02	63.2 ± 0.8	250.1 ± 1.4	-

^a^100 µg/mL

### Antimicrobial screening

The isolated clinical strains were screened for their antibiograms and it was evident that among all tested antibiotics only *Paenibacillus dendritiformis* and *Bacillus paramycoides* and *Bacillus chungangensis* were susceptible to imipenem whereas a borderline sensitivity was recorded against *Bacillus chungangensis* (Table 7). Likewise *Paenibacillus dendritiformis* was sensitive to ciprofloxacin, whereas a border line sensitivity was seen against both isolates of *Bacillus chungangensis.* All other antimicrobial agents were resistant to isolated strains (Table 7).

**Table 7:**
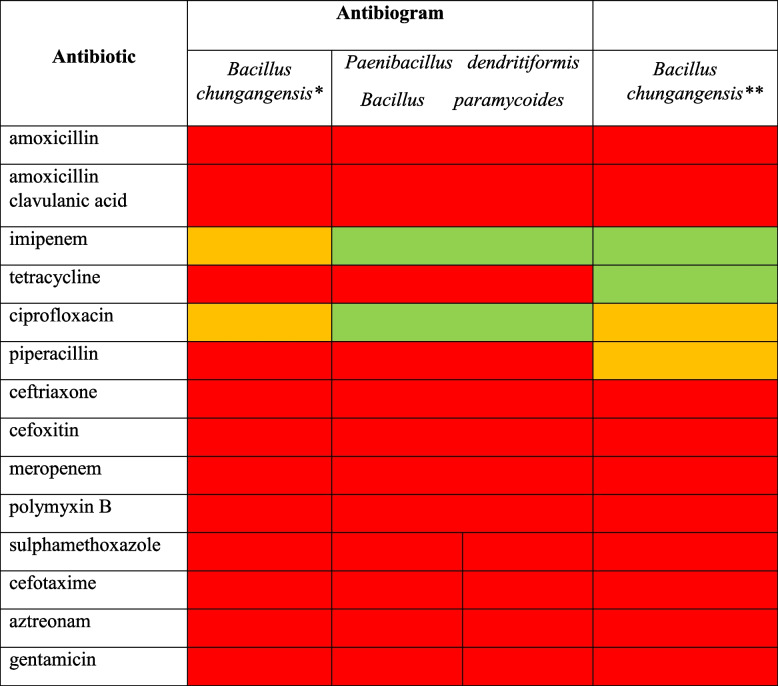
Antibiogram studies of oral pathogens against selected antimicrobial agents

* strain 1

** strain 2

Susceptible (Zone > 13 mm)

Moderate (Zone 10- 13 mm)

Resistant (Zone < 10 mm)

The *C. bergemia* essential oil was analsyed against isolated strains and a moderate inhibition was observed. Amongst tested strains, highest activity (MIC 0.125 mg/mL) was recorded against *Bacillus paramycoides* and *Bacillus chungangensis* (Table 8). In comparative analysis, *C. bergemia* essential was analsyed against standard strains (ATCC) and a significant inhibition was noticed against all tested strains (0.0312–0.0625 mg/mL) (Table 9), that was an indication of prominent activity against resistant strains.

**Table 8:** Determination of MIC against clinically isolated strains

**S.No**	**Clinical Strains**	**Codes**	**MIC (mg/mL)**	**MBC(mg/mL)**
1	*Bacillus chungangensis*	2 M	0.125 ± 0.000	0.125 ± 0.000
2	*Bacillus paramycoides*	4 M	0.75 ± 0.000	1.5 ± 0.000
3	*Bacillus chungangensis*^*a*^	U5	0.125 ± 0.000	0.125 ± 0.000
4	*Paenibacillus dendriformis*	C14	0.25 ± 0.000	0.25 ± 0.000

Standard drug imipenem 64.4 µg/mL against *Bacillus chungangensis, Paenibacillus dendritiformis*, 128.8 µg/mL against *Paenibacillus dendritiformis* and greater that 128.8 against *Bacillus chungangensis*^*a*^*;* values are means of triplicate determination (*n* = 3)

**Table 9:** Determination of MIC against standard strains

**S.No**	**Strains**	**MIC (mg/mL)**	**MBC(mg/mL)**
1	*Klebsiella pneumoniae*	0.0625 ± 0.000	0.125 ± 0.000
2	*E. coli*	0.0312 ± 0.000	0.0312 ± 0.000
3	*Staphylococos aureus*	0.0312 ± 0.000	0.0624 ± 0.000

Standard drug imipenem 44.4 µg/mL against *Klebsiella pneumoniae*, 22.4 µg/mL against all other strains values are means of triplicate determination* (n* = *3)*

### Antibiofilm and anti Q.S activities

**Fig. 8:**
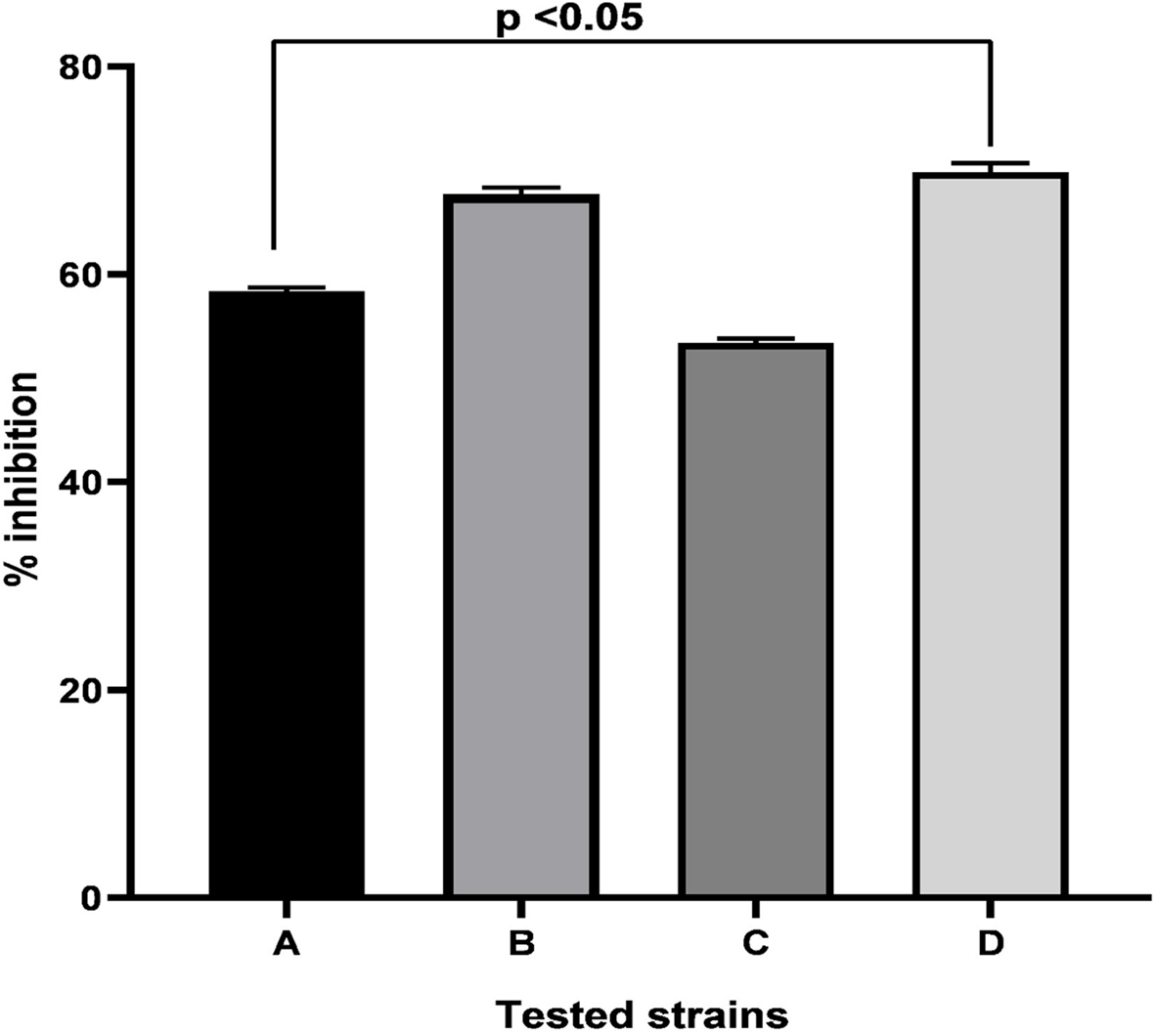
Anti biofilm inhibition assay of *C. bergemia* essential oil against tested strains *Bacillus chungangensis* (**A**), *Bacillus paramycoides* (**B**), Bacillus chungangensis* (**C**), *Paenibacillus dendritiformis* (**D**) tested at 2%(v/w) concencntration

**Fig. 9:**
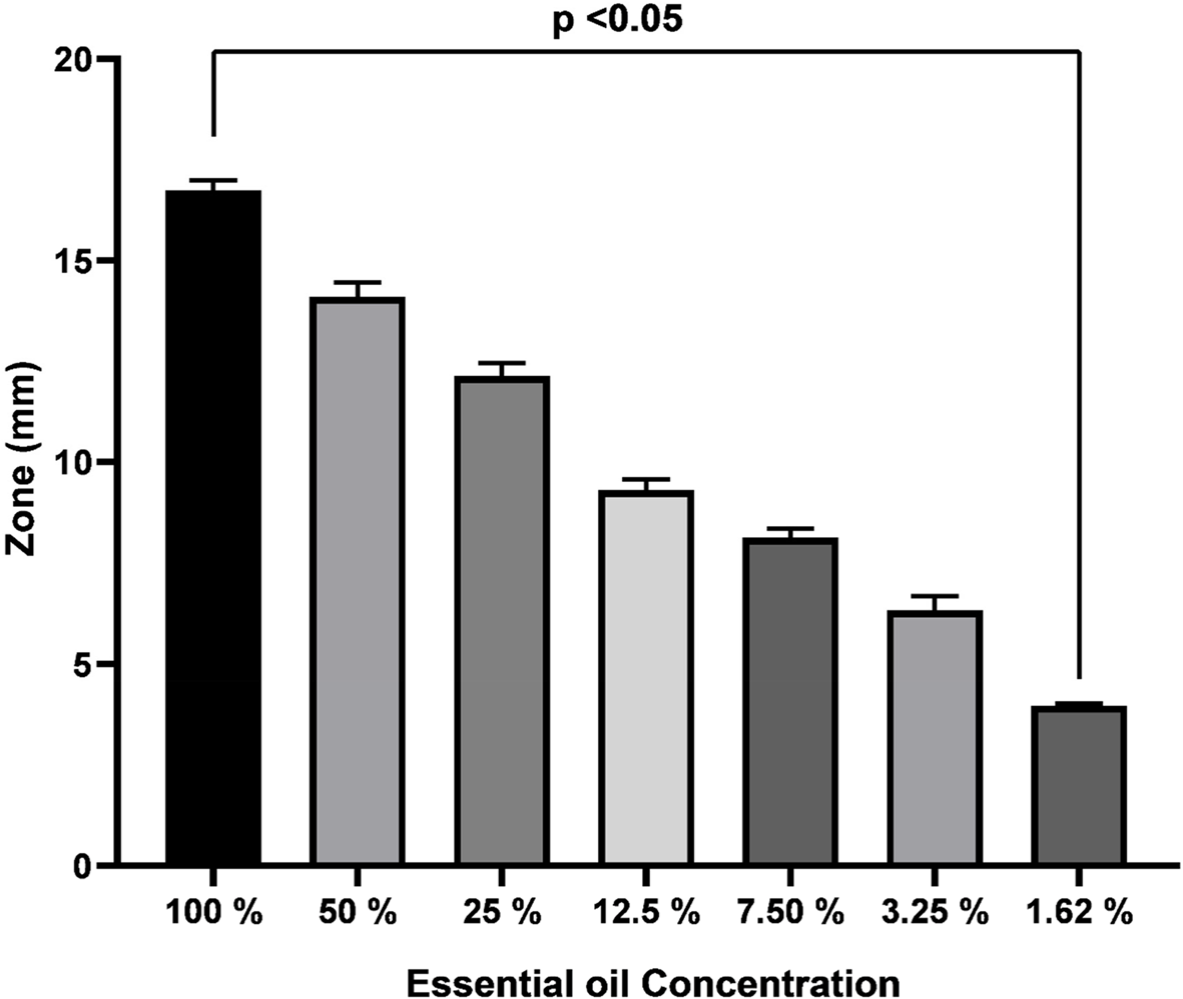
Anti Quorum sensing activities of *C. Bergemia* essential oil

**Fig. 10:**
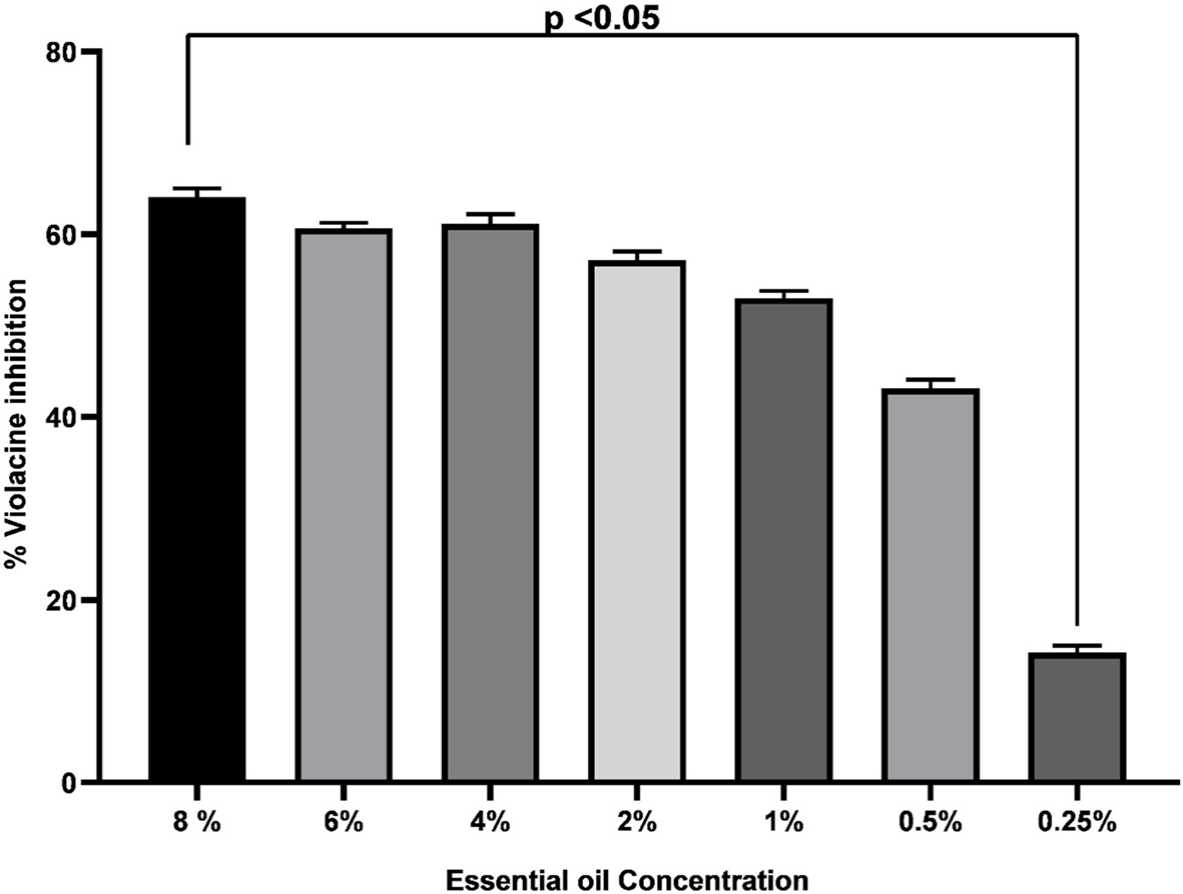
Violacine inhibition assay of *C. Bergemia* essential oil

Our preliminary investigations (congo red agar) confirmed that all clinical strains were biofilm producers, and thus antibiofilm activities were performed. A significant inhibition of biofilm produced by all tested strains was recorded (Fig. 8) with highest inhibition (69.8%) seen in case of *Paenibacillus dendritiformis*. Likewise, C*. bergemia* essential oil showed significant inhibition of *C. vioalceum* at diverse concentration (Fig. 9) that indicated significant antiquorum sensing potential. This was further evident from violacein inhibition assay where a significant inhibition of violacine inhibition was noticed (Fig. 10). Thus the antibiofilm activities of *C. bergemia* can be attributed to anti QS inhibition potential.

## Discussions

*Citrus bergemia* is an important medicinal plant, whose several parts has usage in traditional medicinal systems including Italian, Greece and Chinese [35]. Generally, essential oil from peel of *C. bergemia* is known for anti-inflammatory, antibacterial anticancer, antidiabetic anti-viral properties [36, 37]. We explored flower essential of this plant against oral pathogens, since prevalence rate of such infections is too high [38] and existing therapies (antibiotics) have become resistant. The prevalence rate of such infections is too high in developing countries [39], thus there exists a great potential for new alternative treatments. To best of our knowledge, this is first report exploring potential of *C. bergemia* against oral pathogens.

Component analysis in GC–MS revealed several monoterpenoids including linalool (1,6-Octadien-3-ol,3,7-dimethyl), limonene, *p*-menth-1-ol, 8-ol, aromadendrene, sabinene, β-pinene and β-myrcene, that is comparably diverse in reference to peel essential oil, since several monoterpenoids were discovered from peels including limonene, linalool and β-pinene [40]. The monoterpenes, due to their small M.wt. and specific chemical structures are capable of producing several biological activities [41]. Further, these are categorized as “GRAS” i.e. generally recognized as safe with respect to human health and environment [42]. Thus these can be used as potential therapeutic agents in infection control.

Online computational tools have become a tool of prime importance in drug discovery these days. These tool are based on several algorithms, that enable simulation with reference to targets, and thus provide a prediction with high probability [43]. We investigated major components for their druglikeness, bioavailability and possible antimicrobial targets. As explained earlier, all major components followed lipinski’s rule of five however as light deviations from drug likeness scores were noticed. However, they were minor and may have a little effect on drug bioavailability. Never the less, in limonene, β-myrcin and β-pinene the number of H-bond donors and acceptors were zero, that indicate a limited or no bond formation with target amino acids, thus limiting the biological activity and polarity imbalance that may effect permeability and drug solubility [44]. The pharmacokinetic spectrum analysis revealed that except aromdendrene, all drugs may cross the blood brain barrier and therefore may have effect on CNS. This could possibly be reason that essential oils are effectively used in aromatherapy [45]. Likewise, all parameters of tested compounds followed the cut points of bioavailability radar and therefore regarded as molecules with agreeable bioavailability. cytochrome p450 enzyme in liver are very important for drug metabolism and ADMET analysis indicated that most of tested drug molecules were neither substrate nor enzymes for such enzymes, that show lower metabolism in liver [46]. Once the initial drug likeness and bioavailability parameters checking, we investigated possible antimicrobial potential using PASS analysis. The prediction of activity spectrum of substances is an important tool that indicates probability of possible biological activity [24] that was antimicrobial potential in this case. A high confidence interval in PASS results indicated a good possible antimicrobial activity.

Oxidative stress is a key marker that leads to several diseases including bacterial infections [47]. Several investigations have shown higher oxidative stress in several bacterial infections, that is possibly due to altered metabolic pathways and generation of reactive oxidation products (ROS) during bacterial infections [48]. *C. bergemia* essential oil showed high phenolic contents and it was obvious that it may have strong antioxidant activities [49], as evident from DPPH, H_2_O_2_ and FRAP assays. The essential oils are mainly consisted up of monoterpenoids that participate in a oxidative chemical reaction by react through H atom and inhibition of free radicals chain in reaction [50]. Further compounds with strong antioxidant activity may have strong antimicrobial properties [51].

The *C. bergemia* essential oil presented significant inhibition of resistant strains, that showed potential of this essential oil against oral pathogens. Interestingly the MIC of standard strains against *C. bergemia* was several fold higher. The strong antimicrobial activity was attributed to presence of monoterpenes, that may interacted synergistically. Studies have shown that monoterpenoids due to their lipophilic nature are mainly partitioned from an aqueous phase into bacterial membrane structures [52]. This partitioning effect leads to increase in membrane permeability, destruction of membrane bound structures, interference with ion transport and bacterial cell membrane expansion [53]. Our experiment using congo red assay confirmed that all tested strains were biofilm producers, and thus we investigated *C. bergemia* essential oil for their potential biofilm activities. It was observed that *C. bergemia* showed a significant inhibition of biofilms produced by tested strains by block cell–cell signaling mechanism (quorum sensing) in a dose dependent manner. Bacterial biofilms are includes a matrix of extracellular polymeric substances, that limit the activity of antimicrobial agents to kill bacteria and their permeability to reach target site [54], that leads to antimicrobial resistance. Investigators have shown that monoterpenoids mainly inhibit biofilm formation at early stage by inhibiting formation of flagella [55] blocking biosynthesis of poly-n-acetylglucosamine polymers biosynthesis, that are major elements of bacterial biofilm and quorum sensing [56].

## Conclusion

Oral bacterial infections are important health concern that may lead to development of serious complications of oral cavity. Essential oils are important remedies for ailment of bacterial infections including oral pathogens. We concluded that *C. bergemia* flower essential oil posess significant anti-microbial, antibiofilm and anti Qs activities against oral pathogens including *Bacillus chungangensis*, *Bacillus paramycoides* and *Paenibacillus dendriformis* that can be due strong antioxidant potential. The seasonal variations do effect essential oil concentrations and therefore biological activities too, thus future studies are required to investigate essential yield, collection time and biological activities.

### Supplementary Information

**Supplementary Material 1. **

## Data Availability

All available data is mentioned in the manuscript.

## References

[1] Ben Hsouna A, Ben Halima N, Smaoui S, Hamdi N (2017). *Citrus lemon* essential oil: Chemical composition, antioxidant and antimicrobial activities with its preservative effect against Listeria monocytogenes inoculated in minced beef meat. Lipids Health Dis.

[2] Nauman M C, Johnson JJ. "Clinical application of bergamot (*Citrus bergamia*) for reducing high cholesterol and cardiovascular disease markers. Integr Food Nutr Metabol. 2019;6(2). 10.15761/IFNM.1000249. 10.15761/IFNM.1000249 PMC6497409 31057945

[3] Mabberley DJ (2004). *Citrus* (*Rutaceae*): A review of recent advances in etymology, systematics and medical applications. Blumea J Plant Taxon Plant Geogr.

[4] Bhatia H, Pal Sharma Y, Manhas RK, Kumar K (2015). Traditional phytoremedies for the treatment of menstrual disorders in district Udhampur, J&K India. J Ethnopharmacol.

[5] Russo M, Bonaccorsi I, Costa R, Trozzi A, Dugo P, Mondello L (2015). Reduced time HPLC analyses for fast quality control of citrus essential oils. J Essent Oil Res.

[6] Mannucci C, Navarra M, Calapai F, Squeri R, Gangemi S, Calapai G (2017). Clinical pharmacology of Citrus bergamia: A systematic review. Phytother Res.

[7] Navarra M, Mannucci C, Delbò M, Calapai G (2015). *Citrus bergamia* essential oil: from basic research to clinical application. Front Pharmacol.

[8] Dugo G, Bonaccorsi I. (2014). Citrus bergamia bergamot and its derrivatives. Dugo G, Bonaccorsi I (eds). New York: CRC Press; 2014;51:xvii.

[9] Andrys D, Kulpa D, Grzeszczuk M, Bihun M, Dobrowolska A (2017). Antioxidant and antimicrobial activities of Lavandulaangustifolia Mill. field-grown and propagated in vitro. Folia Hort..

[10] Hyldgaard M, Mygind T, Meyer RL (2012). Essential oils in food preservation: mode of action, synergies, and interactions with food matrix components. Front Microbiol.

[11] Rehman R, Hanif MA, Mushtaq Z, Al-Sadi AM (2016). Biosynthesis of essential oils in aromatic plants: A review. Food Rev Int.

[12] Kokina M, Salević A, Kalušević A, Lević S, Pantić M, Pljevljakušić D, Šavikin K, Shamtsyan M, Nikšić M, Nedović V (2019). Characterization, antioxidant and antibacterial activity of essential oils and their encapsulation into biodegradable material followed by freeze drying. Food Technol Biotechnol..

[13] Pendino G (1998). Il bergamotto in terapia medica: attualità e prospettive. Essenze e derivati agrumari..

[14] Bascones-Martínez A, Figuero-Ruiz E. Periodontal diseases as bacterial infection. Med Oral Patol Oral Cir Bucal. 2004;9 Suppl:101-7; 92-100. English, Spanish. 10.4321/s1699-65852005000300002. 10.4321/s1699-65852005000300002 15580140

[15] Reynolds-Campbell G, Nicholson A, Thoms-Rodriguez CA (2017). Oral Bacterial Infections: Diagnosis and Management. Dent Clin North Americ..

[16] Listl S, Galloway J, Mossey PA, Marcenes W (2015). Global Economic Impact of Dental Diseases. Res Report Clin J Dental Res..

[17] Bond JC, McDonough R, Alshihayb TS, Kaye EK, Garcia RI, Heaton B (2023). Periodontitis is associated with an increased hazard of mortality in a longitudinal cohort study over 50 years. J Clin Periodontol.

[18] Yadav K, Suh KN, Eagles D, MacIsaac J, Ritchie D, Bernick J, Thiruganasambandamoorthy V, Wells G, Stiell IG (2019). Predictors of Oral Antibiotic Treatment Failure for Nonpurulent Skin and Soft Tissue Infections in the Emergency Department. Acad Emerg Med.

[19] Rafey A, Amin A, Kamran M, Haroon U, Farooq K, Foubert K, Pieters L (2021). Analysis of plant origin antibiotics against oral bacterial infections using In vitro and In silico techniques and characterization of active constituents. Antibiotics.

[20] Lipinski CA, Lombardo F, Dominy BW, Feeney PJ (2001). Experimental and computational approaches to estimate solubility and permeability in drug discovery and development settings. Adv Drug Deliv Rev.

[21] Hari S (2019). *In silico* molecular docking and ADMET analysis of plant compounds against IL17A and IL18 targets in gouty arthritis. J Appl Pharm Sci.

[22] Daina A, Zoete V (2016). A BOILED-Egg To Predict Gastrointestinal Absorption and Brain Penetration of Small Molecules. ChemMedChem..

[23] Daina A, Michielin O, Zoete V (2017). SwissADME: a free web tool to evaluate pharmacokinetics, drug-likeness and medicinal chemistry friendliness of small molecules. Sci Rep.

[24] Amin MR, Yasmin F, Hosen MA, Dey S, Mahmud S, Saleh MA, Emran TB, Hasan I, Fujii Y, Yamada M (2021). Synthesis, Antimicrobial, Anticancer, PASS, Molecular Docking, Molecular Dynamic Simulations & Pharmacokinetic Predictions of Some Methyl _-D-Galactopyranoside Analogs. Molecules..

[25] Yu S, Jensen V, Seeliger J, Feldmann I, Weber S, Schleicher E, Häussler S, Blankenfeldt W (2009). Structure elucidation and preliminary assessment of hydrolase activity of PqsE, the *Pseudomonas* quinolone signal (PQS) response protein. Biochemistry.

[26] Chen G, Swem LR, Swem DL, Stauff DL, O'Loughlin CT, Jeffrey PD, Bassler BL, Hughson FM (2011). A strategy for antagonizing quorum sensing. Mol Cell.

[27] Trott O, Olson AJ (2010). AutoDock Vina: improving the speed and accuracy of docking with a new scoring function, efficient optimization, and multithreading. J Comput Chem..

[28] Berman H, Westbrook M, Feng JZ, Gilliland GT, Bhat H, Weissig IN (2005). Discovery Studio Visualizer.

[29] Rajamanikandan S, Sindhu T, Durgapriya D, Sophia D, Ragavendran P, Gopalakrishnan VK (2011). Radical scavenging and antioxidant activity of ethanolic extract of Mollugonudicaulis by in vitro assays. Ind J Pharm Educ Res.

[30] Aryal S, Baniya MK, Danekhu K, Kunwar P, Gurung R, Koirala N (2019). Total Phenolic Content, Flavonoid Content and Antioxidant Potential of Wild Vegetables from Western Nepal. Plants (Basel).

[31] Chandel C, Sharma VK, Rana PS, Dabral M, Aggarwal S, Saklani P (2020). Assesment of antimicrobial and antioxidant potential of cytoplasmic male sterile lines of pepper. SN Appl Sci.

[32] Weseler AHKR, Geiss HK, Saller R, Reichling JJDP (2005). A novel colorimetric broth microdilution method to determine the minimum inhibitory concentration (MIC) of antibiotics and essential oils against Helicobacter pylori. Pharmazie.

[33] Labbate M, Queck SY, Koh KS, Rice SA, Givskov M, Kjelleberg S (2004). Quorum sensing-controlled biofilm development in Serratia liquefaciens MG1. J Bacteriol.

[34] Husain FM, Ahmad I, Al-Thubiani AS, Abulreesh HH, AlHazza IM, Aqil F (2017). Leaf Extracts of Mangifera indica L. Inhibit Quorum Sensing - Regulated Production of Virulence Factors and Biofilm in Test Bacteria. Front Microbiol..

[35] Navarra M, Mannucci C, Delbò M, Calapai G (2015). Citrus bergamia essential oil: from basic research to clinical application. Front Pharmacol.

[36] Risitano R, Currò M, Cirmi S, Ferlazzo N, Campiglia P, Caccamo D (2014). Flavonoid fraction of Bergamot juice reduces LPS-induced inflammatory response through SIRT1-mediated NF-κB inhibition in THP-1 monocytes. PLoS ONE.

[37] Mollace V, Sacco I, Janda E, Malara C, Ventrice D, Colica C (2011). Hypolipemic and hypoglycemic activity of bergamot polyphenols: from animal models to human studies. Fitoterapia.

[38] DelleMonache S, Sanità P, Trapasso E, Ursino MR, Dugo P, Russo M (2013). Mechanisms underlying the anti-tumoral effects of *Citrus **Bergamia* juice. PLoS ONE.

[39] Jain N, Dutt U, Radenkov I, Jain S (2023). WHO's global oral health status report 2022: Actions, discussion and implementation. Oral Dis.

[40] Melliou E, Michaelakis A, Koliopoulos G, Skaltsounis AL, Magiatis P (2009). High quality bergamot oil from Greece: Chemical analysis using chiral gas chromatography and larvicidal activity against the West Nile virus vector. Molecules.

[41] Mondello F, De Bernardis F, Girolamo A, Salvatore G, Cassone A (2003). In vitro and in vivo activity of tea tree oil against azole-susceptible and-resistant human pathogenic yeasts. J Antimicrob Chemother.

[42] Medina-Franco JL, Martínez-Mayorga K, Peppard TL, Del Rio A (2012). Chemoinformatic Analysis of GRAS (Generally Recognized as Safe) Flavor Chemicals and Natural Products. PLoS ONE.

[43] Dias R, de Azevedo WF Jr (2008). Molecular docking algorithms. Curr Drug Targets..

[44] Kenny PW (2022). Hydrogen-Bond Donors in Drug Design. J Med Chem..

[45] Soares GABE, Bhattacharya T, Chakrabarti T, Tagde P, Cavalu S (2021). Exploring Pharmacological Mechanisms of Essential Oils on the Central Nervous System. Plants (Basel)..

[46] Flores-Holguín N, Frau J, Glossman-Mitnik D (2021). Computational Pharmacokinetics Report, ADMET Study and Conceptual DFT-Based Estimation of the Chemical Reactivity Properties of Marine Cyclopeptides. ChemistryOpen.

[47] Spooner R, Yilmaz O (2011). The role of reactive-oxygen-species in microbial persistence and inflammation. Int J Mol Sci..

[48] Ivanov AV, Bartosch B, Isaguliants MG (2017). Oxidative Stress in Infection and Consequent Disease. Oxoid Med Cell Longev.

[49] Kähkönen MP, Hopia AI, Vuorela HJ, Rauha JP, Pihlaja K, Kujala TS, Heinonen M (1999). Antioxidant activity of plant extracts containing phenolic compounds. J Agric Food Chem.

[50] Atere CT, Ge TD, Zhu ZK, Liu SL, Huang XZ, Shibsitova OL, Guggenberger G, Wu JS. Assimilate allocation by rice and carbon stabilisation in soil: effect of water management and phosphorus fertilisation. Plant Soil. 2019;445:153–67.

[51] Baharfar R, Azimi R, Mohseni M (2015). Antioxidant and antibacterial activity of flavonoid-, polyphenol- and anthocyanin-rich extracts from Thymus kotschyanusboiss&hohen aerial parts. J Food Sci Technol.

[52] Sikkema J, de Bont JAM, Poolman B. Interactions of cyclic hydrocarbons with biological membranes. J Biol Chem.1994;269:8022–8. 8132524

[53] Badawy MEI, Marei GIK, Rabea EI, Taktak NEM (2009). Antimicrobial and antioxidant activities of hydrocarbon and oxygenated monoterpenes against some foodborne pathogens through in vitro and in silico studies. Pest Biochem Physiol.

[54] Faleiro ML (2011). The mode of antibacterial action of essential oils. Sci Against Microb Pathog Commun Curr Res Technol Adv..

[55] Okoh SO, Iweriegbor BC, Okoh OO, Nwodo UU, Okoh AI (2016). Bactericidal and antioxidant properties of essential oils from the fruits Dennettiatripetala G. Baker BMC Complement Altern Med.

[56] Cáceres M, Hidalgo W, Stashenko E, Torres R, Ortiz C (2020). Essential Oils of Aromatic Plants with Antibacterial, Anti-Biofilm and Anti-Quorum Sensing Activities against Pathogenic Bacteria. Antibiotics (Basel)..

