# Supplementary material for: Potential role of Citrus bergamia flower essential oil against oral pathogens
Source: BMC Complement Med Ther. 2024 Apr 12;24:157. doi: 10.1186/s12906-024-04457-7 (PMC11010433; doi:10.1186/s12906-024-04457-7)
Supplement: Supplementary file 1 — Supplementary Material 1. [file 12906_2024_4457_MOESM1_ESM.docx]

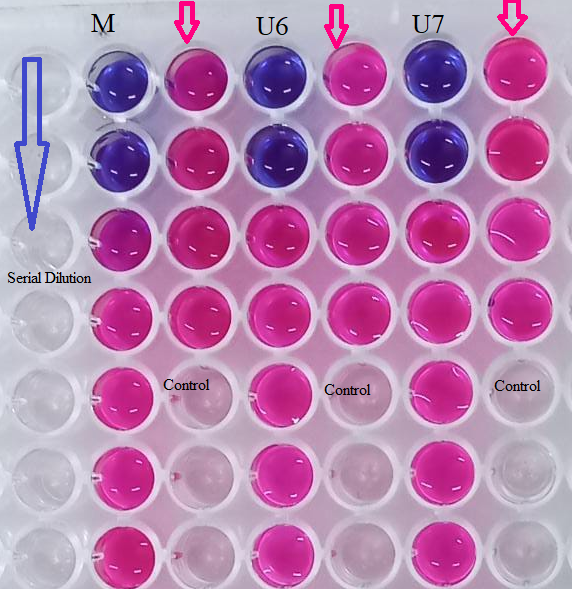

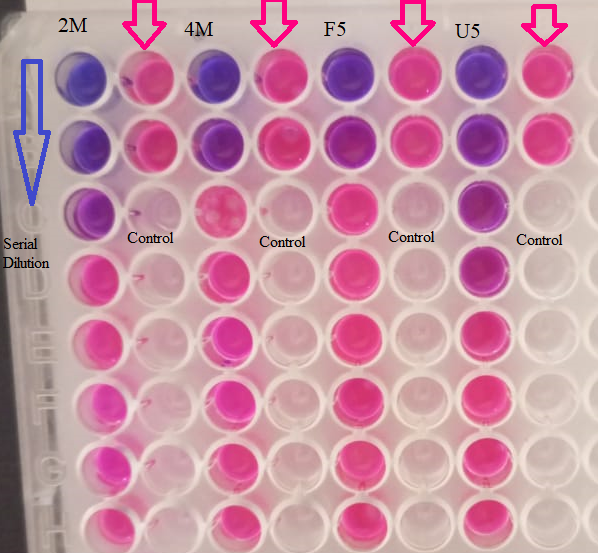


Figure S1: 96 microplate resazurin based assays for determination of MIC

A


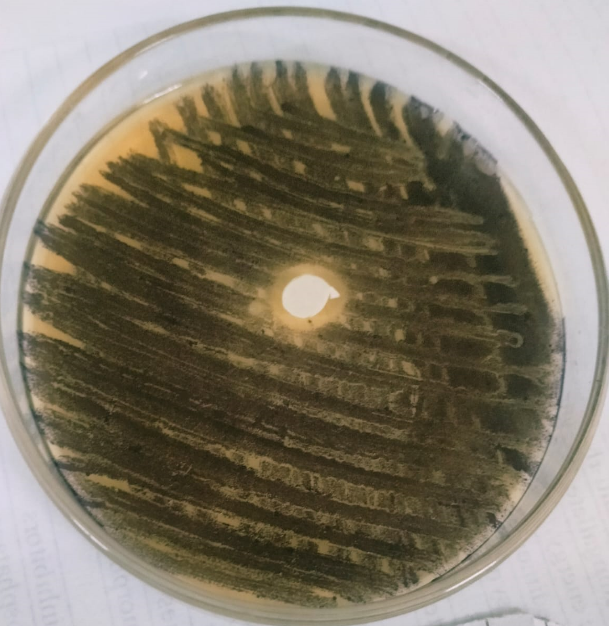


B


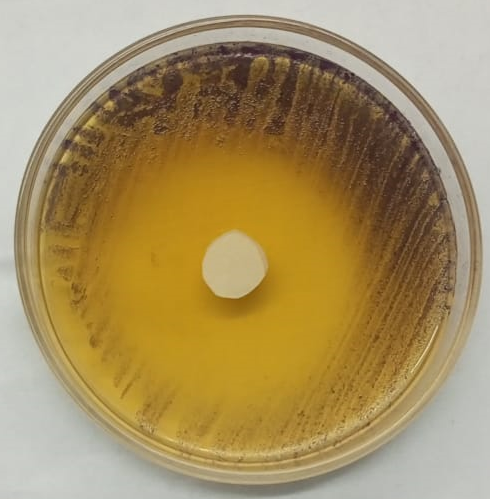


Figure S2: Antiquorum sensing activity of *C. bergemia* Essential oil A (100%) and B (3.25%).
